# Medical Benefits for Nursing Staffs

**Published:** 1913-02-22

**Authors:** 


### NATIONAL INSURANCE ACT DEPARTMENT.

### XLIV.—Medical Benefits for Nursing Staffs.

The question of medical benefit under the National Insurance Act as applied to hospital nursing staffs is one of the difficult matters that cannot be satisfactorily dealt with under the Act as it at present stands. In the past the nurses have almost invariably, if not always, received medical attention and treatment from the staff of the hospital. Nurses have been engaged under a system which by custom provides them with medical attention. Medical treatment, board, lodging, and salary have been all part of the attractions to nurses to enter into the employment of the hospitals. Hospital nurses, therefore, constitute a special class of insured persons for whom the standard benefits of the Act—at least in the matter of medical benefit—are not strictly applicable. They were already provided with medical treatment, and there was therefore no necessity to make the same—or perhaps a less adequate—provision for them from another source.

The matter was not entirely overlooked when the Bill was before Parliament, and the "Harmsworth" amendment was introduced partly in the interests of the nurses, and we shall endeavour to show that measures should be taken at once to obtain the full benefit of that amendment.

Public attention has again been called to the question by the striking letter from Mr. Paterson which appeared in the *Times* for last Tuesday week, to which we referred briefly in another column in our last week's issue. Mr. Paterson did valiant service for the nurses in calling attention to their special circumstances when the Bill was before Parliament, and we understand that he received an intimation that the substance of his plea would be recognised on the Report stage of the Bill. Unfortunately, the amendments suffered by reason of the guillotine, and the present position is the direct consequence of stifled debate.

It is important that the exact position should be grasped, and that the various remedies and alternatives provided by the Act should be closely examined before passing adverse criticism. The examination may also enable us to put forth a useful suggestion for an amendment.

### Who Pays for Medical Benefit?

Let us take the normal case of a nurse receiving a salary of, say, £25 a year, with board, lodging, and medical treatment provided free. She must be insured under the Act, and contributions amounting to 6d. a week are paid on her behalf. Half of this sum—namely, 3d. a week—is deducted from her salary, making a total sum of 13s. a year if she is in full employment throughout the year. In a similar way the employer—the hospital authority—pays 13s. a year. Amongst the benefits provided by this payment is medical treatment and attendance, the cost of which, as included in the contributions, is 6s. a year—the additional sum now promised to the doctors being a special Government grant made from monies voted by Parliament for the purpose.

It is clear, therefore, that medical benefit is being paid for, and those who pay for it are entitled in common justice to reap the advantage of their payments.

Now there arises a problem in apportionment. It might be claimed by the hospital authorities that as they pay more than the cost of the medical treatment that the whole of the benefit is provided out of their contribution. If that contention be upheld they would have the right of appropriating the amount received for the treatment actually given to the nurses. On the other hand, it might be claimed by the nurses that they now pay for their medical treatment, and that some corresponding advantage should be accorded to them in view of the fact that they were formerly entitled to the treatment as part of their remuneration.

Our own view is that it is impossible to apportion the whole of the cost either to the hospitals or nurses, and that the payments of contribution must be looked upon as a whole, each constituent bearing its proportion of the cost of all the benefits—medical, sickness, disablement, maternity, and sanatorium.

In these circumstances it appears to us that neither the nurses nor the hospital authorities have any special claim to receive the monetary value of the medical benefit, and that if a division were actually required it should be made in equal shares. They should, however, agree between themselves to see that advantage is obtained from the payments for medical benefit included in their contributions.

### The Panel System not Suitable.

The ordinary method by which the benefit is administered is through a panel doctor. Every nurse has the right, under the Act, of selecting any doctor on the panel, but a difficulty would arise if none of the hospital staff have joined the panel. It might be possible, of course, in such case for the nurses to make their own private arrangements with the staff, but the Insurance Committees have adopted the attitude that permission can only be granted to insured persons to make their own arrangements with doctors off the panel in special circumstances, and it is thought hardly likely that many Insurance Committees would allow the nurses freedom of choice outside the panel. The demand for such private arrangements might, in fact, be considered a favourable opportunity for coercing still more doctors to join the panel.

Should Hospitals Become Approved Institutions?

Another method which should be available in most cases is that indicated in Section 15, sub-section 4, commonly known as the "Harmsworth" amendment. By this it is provided that the regulations of the Insurance Commissioners for the administration of medical benefit shall allow medical treatment and attendance under any system or through any institution existing at the time of the passing of the Act (December 16, 1911) to be treated as, or as part of, medical benefit under the Act. This provision is subject, however, to the approval of the system or institution not only by the Insurance Committee, but also by the Insurance Commissioners, and it is further expressly stipulated that such arrangements cannot be allowed to deprive the insured person of the right of selecting the practitioner by whom he (or she) wishes to be attended and treated.

The vast majority of the hospitals would seem to come under the definition of institutions existing at the time of the passing of the Act, and they should, therefore, be able to obtain the advantages conferred by the sub-section. We hear, however, that in one case at least approval has been refused. On what grounds the refusal was made we are unable to say, and it may be that there were special circumstances which made it necessary. It is, perhaps, unwise to generalise on the strength of this case, but the attitude of the Insurance Committees should be that refusal is only given when it is manifest that the approval of the institution is absolutely undesirable.

The regulations of the Commissioners stipulate that approval shall not be granted unless the Committee are satisfied that the treatment is adequate, and that every insured person obtaining benefit under the scheme is entitled on giving due notice to free choice in the matter without incurring any pecuniary loss or other penalty. A further condition of approval is that the institution must furnish periodical accounts and returns as required by the Committee with the consent of the Commissioners. These regulations appear to be essentially reasonable, and if the hospital authorities and nurses act in concert it would seem that favourable consideration should be accorded to applications by the institutions for approval.

Seeing that the hospitals would receive under this system the grant for medical benefit for those of its nursing staff who elected to receive treatment in the institution, and that formerly it gave such treatment without payment, it would be only just that some additional advantage should be given to the staff of a corresponding value. The same may be said in regard to those exceptional cases where the nurses elect to be treated by practitioners not on the medical staff of the hospital. The hospital will in both cases make some small saving, and advantage of this should be given to the nurses concerned.

The most useful suggestion we have to offer is that the sums so available should be pooled and used for the dental treatment of those nurses in need thereof so far as the fund will go. It has to be remembered that the fund will be comparatively small, and that its application to more ambitious schemes is impracticable, but this is no reason why full advantage should not be taken of the amount at disposal.

A Suggested Amendment.

The most obvious corrective for the whole situation is that nurses in hospitals should be allowed to contract out of the medical benefit of the Act, thus reducing their contribution by 1½d. a week. Unfortunately, the Insurance Commissioners are not able to treat a question like this as a business proposition in the same way as a private corporation, which adapts its terms to the requirements of its customers. The Commissioners are bound by the Act, and at present they have no power to make such an alteration in the contributions in consideration of the relinquishment of benefit.

Our suggestion is that an amendment framed to meet the requirements of the nurses on the lines indicated should be brought forward and pressed for inclusion in an amending Act on the first available opportunity. There are certain obvious difficulties in the way of such an arrangement. Difficulties in still further complicating the already very complicated accounts of the approved societies, and in making the conditions apply only so long as the nurses are actually in hospital service. These difficulties are not, however, insuperable, and we can see no reason why the nursing profession should not receive the same consideration for their special conditions of service as was accorded, in a different way, and to meet other special requirements, in the case of elementary school teachers.

**NOTE.—Questions and Correspondence on all doubtful points are invited, and should be addressed to the Editor, The Hospital, 28, 29 Southampton Street, Strand, London, W.C., and marked "Insurance."**

